# Seasonal Variation in the Fate of Seeds under Contrasting Logging Regimes

**DOI:** 10.1371/journal.pone.0090060

**Published:** 2014-03-10

**Authors:** Marina Fleury, Ricardo R. Rodrigues, Hilton T. Z. do Couto, Mauro Galetti

**Affiliations:** 1 Departamento de Ecologia, Universidade Estadual Paulista, Rio Claro, São Paulo, Brazil; 2 Laboratório de Ecologia e Restauração Florestal, Departamento de Ciências Biológicas. Universidade de São Paulo, USP/ESALQ, Piracicaba, São Paulo, Brazil; 3 Laboratório de Métodos Quantitativos, Departamento de Ciências Florestais, USP/ESALQ, Piracicaba, São Paulo, Brazil; University of Marburg, Germany

## Abstract

Seed predators and dispersers may drive the speed and structure of forest regeneration in natural ecosystems. Rodents and ants prey upon and disperse seeds, yet empirical studies on the magnitude of these effects are lacking. Here, we examined the role of ants and rodents on seed predation in 4 plant species in a successional gradient on a tropical rainforest island. We found that (1) seeds are mostly consumed rather than dispersed; (2) rates of seed predation vary by habitat, season, and species; (3) seed size, shape, and hardness do not affect the probability of being depredated. Rodents were responsible for 70% of seed predation and were negligible (0.14%) seed dispersers, whereas ants were responsible for only 2% of seed predation and for no dispersal. We detected seasonal and habitat effects on seed loss, with higher seed predation occurring during the wet season and in old-growth forests. In the absence of predators regulating seed-consumer populations, the densities of these resilient animals explode to the detriment of natural regeneration and may reduce diversity and carrying capacity for consumers and eventually lead to ecological meltdown.

## Introduction

Forest fragmentation, selective logging and defaunation are main drivers of global environmental change that modify biodiversity and environmental conditions in several tropical forests [1]–[3]. At present, anthropogenic change is affecting all tropical forests, and secondary and logged forests are increasingly replacing old-growth forests [4]. Hence, there has been an increasing initiative to promote ecological restoration in tropical forests [5]. The gap in knowledge regarding both the basic functions of fragmented, logged, or degraded secondary forest and the role of invasive plant and animal species in tropical natural ecosystems has resulted in many failed past efforts to regenerate forests [6], [7].

According to the framework of successional mechanisms [8], the best-fitted conceptual model of succession to tropical ecosystems [9], all of the specific factors and processes that affect species availability have equal importance and are components of succession, including the role of animals as an element affecting the progress of succession. Site characteristics and specific parameters must be considered when translating general theoretical statements to useful testable predictions relevant to a particular area, which can later be refined and applied across larger geographic areas for a full successional theory [8]. Despite its ecological relevance, a strong understanding of the basic functions of modified Atlantic rainforest habitats and of the effects of animal composition and distribution on ecosystem processes and resilience remains limited [10], thus preventing the development of a stronger theoretical base for ecological restoration [11].

Forest recovery or advanced regeneration of disturbed areas may be limited by the availability of seeds, as has been suggested for most tropical plants [12], [13]. Seed availability is determined by the presence (soil seed bank), gains (seed rain), and losses (unsafe sites and seed predation) of seeds [14]. Although plants depend on animals as seed dispersers [13], [15], [16], and the factors that may affect the gains by seed rain [17]–[19] are well recognized, nearly all (>99%) seeds primarily dispersed are destined for failure [13]. Thus, seed predators are expected to act as a selective filter, determining which species are able to establish during forest succession, and to have an important impact on most tropical plant species; however, the role of resilient forest-dwelling animals on post-dispersal seed fate remains elusive.

Ants and rodents are resilient animals that have relatively generalized diets and that can both prey upon and disperse seeds, serving as substitute seed dispersers of at least several species in Neotropical zones [16], [20]–[22]. However, in the absence of top-down force regulating, the densities of these resilient animals expand with negative consequences for natural regeneration, and thus, producers impose regulation from the bottom-up reducing diversity and carrying capacity for consumers in a phenomenon called “ecological meltdown” [23].

Different seed predators usually share the same environment and may even overlap in terms of seed species consumed [24], but contrasting habitats could affect the behavior of seed consumers and alter seed dispersal and predation [25]–[27]. An understanding of these species' behavior as it pertains to seed loss is vital for predicting the potential consequences of habitat degradation on plant [28] and forest regeneration [29].

The goal of this study was to examine the factors influencing the seed fate of tropical trees across a disturbance gradient produced by different logging regimes in a rainforest fragment. Specifically, we evaluated the relative importance of rodents (small- and medium-sized mammalian seed eaters) and ants as seed predators, and defined the relationship between key habitat characteristics and the seed fate between seasons (austral summer and winter). We expected to observe higher levels of seed predation, or alternatively seed caching, by rodents than by ants due to differences in the foragers' abilities to handle and move seeds. Consequently rodents will remove a greater total number of seeds than ants. Rodents and ants may differ in their susceptibility to local changes in environmental conditions, differentially affecting their abundance and behavior resulting in predictable suites of species associated with different disturbance regimes [30], and therefore, predictable rates of seed losses among habitats and seed species occur. If resources vary spatially within and among habitats and between seasons [31], we expect that seed losses should also vary between seasons. Along transects, the key vegetation variables that could influence the habitat preferences of seed predators were recorded. The identity of seed predators and seed fate were determined by following the route, final destination and seed marks of seed consumers using the spool-and-line protocol [32]. The results of this study not only help us to better understand a crucial process for rainforest regeneration but also contribute to our knowledge on the basic functions of human-modified Atlantic rainforest habitats, which in turn facilitates the development of conservation and/or ecological restoration tools.

## Materials and Methods

### Study site

The study was conducted on Anchieta Island (45°02′W; 23°27′S), an 806 ha protected area of land in southeast Brazil. The Technical Scientific Committee (COTEC) from Instituto Florestal (IF-SP) issued all of the required permits for the work conducted in Anchieta.

Europeans displaced the original indigenous inhabitants of Anchieta Island, who engaged in subsistence activities, in the mid-19th century [33]. In 1904, the nearly 412 families residing on the island were transferred to the continent when a state prison was founded in the area [33]. During the prison periods (1904–1914 and 1928–1952), farming and selective logging resulted in land-use and land-cover changes on Anchieta Island [33]. In 1977, Anchieta Island was converted to a State Park with a strict regime prohibiting human intervention. Although the island is approximately 400 m from the mainland – a surmountable obstacle for several animals and propagules (see review by [34] and references therein) – and was left for 36 years to regenerate naturally, the island is largely covered by successional habitat patches resulting from differential land-use intensity.

Our surveys and experiments were conducted across the three habitats (old fields, early-secondary forests, and old-growth forests) on the island, which comprise 93% of the total area. The early-secondary forest (409 ha, 50.7% of total land cover) is predominantly composed of small trees and is characterized by a broken canopy with a high density of anemochorous and pioneer species in the seed rain [35]. Palm trees, a key component of tropical forests [36], are present in early-secondary forest but are more common in the 211 ha of old-growth forest (26.1%). This stage is characterized by more stratified vegetation, including epiphytes, and the highest canopy cover. The 135 ha of old fields are composed of herbaceous species with sparse shrubs and patches of bare soil, largely dominated by *Miconia albicans*, *Myrsine coriacea, M. umbellata*, and fern (*Gleichenella* spp.); this area has the lowest canopy cover. Human settlements, offshore rocks, sandy beaches, and sandy coastal forests cover the remaining 51.5 ha of the island.

With only three ground-dwelling seed consumers found on the island, the diversity of small- and medium-sized rodents in Anchieta is low compared to that of other tropical forests [37]–[41], probably resulting from past extinctions of small mammals. The long history of forest disturbance, lack of predators and recent introduction of mesopredators probably impoverished the small mammal community [43]. Previous studies have reported two small-sized rodents, the Ihering's Atlantic spiny rat (*Trinomys iheringi*) and the black-footed pygmy rice rat (*Oligoryzomys nigripes*) [43], and one medium-sized red-rumped agouti (*Dasyprocta leporina*) [42]. The spiny rat *T. iheringi* is less abundant here than in other Atlantic rainforest sites, whereas the rice rat *O. nigripes* [43] and the agouti [42] reach high densities. In the studied area, the habitat distribution and abundance of rodents are not randomly distributed: *O. nigripes* is more abundant in the old fields, while *T. iheringi* is more common in the old growth forest [43]. The agouti, a deliberately introduced species but probably already present in the area, is rarely sighted in old fields, and reaches high abundances in early-secondary and old-growth forests ([42]; R.S. Bovendorp, pers. comm.). The species-area curve predicts from three to five rodents already been extirpated from the Anchieta [43] due to direct and indirect anthropogenic effects.

### Seed selection and preparation

We tested four tree species with different seed dispersal modes – dispersal by small- and large-gaped birds, mammals, and wind – and different successional stages (Table 1). Although differential seed predation by ants and mammals includes the full range of seed sizes in the community, our comparison focused only on seeds larger than 20 mg. Some studies have demonstrated a trend of higher seed losses in species with smaller seeds [44]–[46], but we have focused on seeds large enough to be preyed upon by both groups because (i) rainforests contain more large-seeded species than any other type of forest [47]; (ii) large-seeded species are often preferentially cached by seed predators [48], [49]; and (iii) large-seeded species have a high potential for survival in disturbed habitats if they survive through germination [50]. Plant species were selected based on local abundance and seed availability at the time of the study. Ripe fruits and pods were collected from several trees in the study site area. The seeds were cleaned of pulp and visually inspected for insect infestations. We discarded (i) seeds identified as potentially damaged, decayed, or desiccated; (ii) those with emerging radicles or abnormal development; and (iii) those with potential bruchid infestation ([17], but see [51]). We saved a random subsample of the collected seeds to quantify functional traits (Table 1).

**Table 1:** Characteristics of the four Anchieta Island tree species utilized in the seed fate experiment, listed in order of increasing shade tolerance.

Species	Family	Habitat	Dispersal syndrome	Seed Mass (g ± SD)	Seed Shape (Shape ± SD)	Relative force (N*cm ± SD)	Total Force (N ± SD)
*Myrsine coriacea*	Primulaceae	Pioneer	Bird (small gape)	0.016±0.004	0.001^−1^±0.000	204.45±52.48	5.79±1.29
*Schizolobium parahyba*	Leguminosae	Early-secondary	Wind	1.740±0.250	1.973±0.810	174.76±49.95	279.68±77.98
*Syagrus romanzoffiana*	Arecaceae	Late-secondary	Mammal	1.631±0.422	0.110±0.050	133.81±30.35	165.13±34.15
*Euterpe edulis*	Arecaceae	Climax	Bird (large gape)	1.111±0.150	0.004±0.006	26.12±5.94	29.83±6.91

Habitat was determined according to Budowski [103]; seed shape is the variance in seed length, depth and width; the total force is the force required to penetrate each seed coat, expressed in Newtons (N); and the relative force (N*cm) is the force required for the animal to access the endosperm, considering the width of the seed opening. Data are expressed as the mean ± the standard deviation.

### Experimental design

The seed fate experiments were primarily conducted along previously established trails across the Anchieta Island; a minimum length of ∼1.5 km per habitat was evaluated.

#### Seed removal and seed fate

We assessed the fate of seeds by applying the spool-and-line protocol [32]. This protocol involves a thread-filled bobbin from which the line is supplied. The seed, drilled and attached to the end of the line, is carried away, allowing the animal route, seed location and fate to be determined [32]. With the exception of *Myrsine coriacea*, whose smaller seed size required quick bonding gel glue to attach the threads, each seed was threaded with a 35 m line spool bobbin fixed parallel to the soil by an L-shaped steel rod, allowing the line to easily unroll when the seed was removed. The spool-and-line protocol is considered an efficient method for describing the fate of seeds consumed by ground-dwelling animals [32].

A total of 60 experimental seed-removal stations were simultaneously established, with 20 stations per habitat. The experimental stations were established at 50 m intervals along the existing trails, and each station consisted of four (15×15-cm) plots ∼1 m from one another. Each plot contained three seeds from a single species gathered from the forest ground to mimic the natural dispersal of seeds by either animals or wind. All seeds within a 2 m radius from the experimental stations were removed from the soil surface before setting up the experiment. Across dry and wet seasons, we determined the fate of 1440 seeds in total (360 seeds/species).

Seeds were set out at each experimental station to determine the fate of seeds in open field, early-secondary, and old-growth forests during both the wet (February–March 2007) and the dry seasons (August–September 2007). After 30 d, a period sufficient to evaluate seed removal in an Atlantic rainforest [21], [52], the number of seeds present that were intact, preyed upon by insects or rodents, moved and buried, or moved and left on the surface were recorded. We considered seeds to have been moved if they were located at least 1 m away from the plot center. Damaged seeds were assumed to have been preyed upon, and marks or other visual signs were used to identify the seed predator. Signs of mammal seed predation included piles of husks, baited seeds in a triangular or quadrangular format and partially eaten seeds with teeth marks. Insect predators, for example, ants of the genus *Atta*, left an empty seed coat. Dispersed seeds were classified as non-cached (dispersed on the forest floor) or scatter-hoarded (cached in the soil or beneath the leaf litter). When the line was broken and a seed was missing, the surrounding area was searched. All but 11 of the seeds set out were either relocated or showed signs of seed predation. Thus, even for seeds that could not be located, seed removal was referred to as seed predation.

#### Seed traits

A subsample of 30 diaspores of each target species (*Euterpe edulis*, *Syagrus romanzoffiana*, *Myrsine coriacea* and *Schizolobium parahyba*) was measured to determine seed mass, shape and hardness. Cleaned seeds (without pulp or wings) were weighed on a microbalance. Then, length, depth and width were measured using an Avenger digital caliper. Seed shape was described as the extent to which the shape differed from sphericity, as determined by variance in seed length, depth and width. This variance had a minimum value of zero in perfectly spherical seeds and a higher value in needle or disc-shaped seeds [53].

Seeds were subjected to a resistance test using a Losenhausenwerk hydraulic testing apparatus at Escola Superior de Agricultura “Luiz de Queiroz” of Universidade de São Paulo, ESALQ/USP. Rodents were the main seed predators on the island, so a sharp steel piece was attached to the equipment to mimic an incisive tooth bite. We recorded the force required to penetrate each seed coat and the width of the seed where it was opened to calculate the total and relative forces required for the animal to access the endosperm, expressed as Newtons (N/species) and Newton centimeters (N*cm), respectively. We analyzed the total and relative forces to examine whether the total force that must be applied by a seed predator was a limiting factor for each tested species or whether the relative force is more important.

#### Site characteristics

Variation in seed losses among habitats has been attributed to differences in vegetation structure [24 and references therein], [26], resource availability and distance to important fruiting trees [54]–[57], and litter thickness [58]–[60]. Thus, seasonal habitat variation among these attributes may reflect the abundance and spatial distribution of microsites preferred by generalist and resilient seed consumers [61].

Vegetation structure: Measures of relative plant abundance, which included the basal area for the overstorey and the cover for the understorey, were obtained by measuring the fraction of the sky visible beneath the canopy (DIFN) and the leaf area index (LAI), respectively. Data were acquired using an LAI-2000 Plant Canopy Analyzer (LI-COR Inc., USA) and were based on indirect measurements determined by calculating the efficiency of light interception by the plant canopy [62].

Plant phenology and distribution of palm trees: The Fournier index (FI) of intensity, a semi-quantitative measurement (scaling from 0 to 4), was applied to score the density of ripe and unripe fruits/seeds in each habitat [63], [64]. The FI was determined at the beginning of the study and on the 30^th^ day after setting up the seed predation experiments for all of the trees with diameter at breast heigh (dbh) ≥15 cm and a distance of ≤2 m from each side of the trail [63], [64]. Due to the importance of palms on Anchieta Island, particularly to the frugivore community [65], we counted the number of palm trees within 4 m of each side of the trails in each habitat.

Litterfall: The effect of leaf litter deposition on seed predation was tested because some authors have noted that rodents' ability to find seeds in plant litter is reduced [58], [59], which increases seed survival and germination [60], whereas ants' ability to find seeds may increase with litter density [66], [67]. The litterfall was sampled in 50×50 cm parcels haphazardly selected in each station (n = 60). The samples were dried at 60°C for 72 h and weighed.

### Data analysis

Two-way ANOVAs with interaction terms were used to test whether seed removal varied according to habitat, plant species and season. Tukey's HSD multiple comparisons procedure was used to separate habitat groups when either habitat or species was found to be a significant factor. The dependent variable (seed predation) was square-root transformed, and the residuals were examined to test the assumptions of normality and homogeneity of variances, respectively [68]. Seed predation was quantified as the proportion of preyed on seeds tracked in each plot. The proportions of depredated seeds per species were analyzed to calculate the main effects of seed predation [69].

Nonmetric multidimensional scaling (NMDS), a multivariate ordination analysis that achieves clear representation of the distance among objects [70], was used to identify the underlying ecological parameters that influence the distribution of seed predation among habitats and species, as well as between seasons. The NMDS ordination followed the recommended procedures [70] and was applied to the site characteristics (LAI, DIFN, litterfall biomass, number of palm stands, production (FI %) of ripe and unripe fleshy fruits) and to the seed traits (seed mass, seed shape, seed hardness expressed as force (N) and relative force (N*cm) to crack the seed), using the arithmetic means for all data.

The NMDS analyses were performed in R, using the metaMDS function ( ) from the vegan package. Euclidian distances were applied between object points and 1,000 random starts to determine the best possible solution. The NMDS biplot was constructed using two dimensions. For instance, Sheppard diagrams were used to inspect the residuals of the NMDS solutions and transformations, as well as the stress values as goodness-of-fit [71]. In addition, the goodness-of-fit of both ordinations was measured as the *R^2^* value of either the linear or non-linear regression of the NMDS distances on the original plots. Spearman's rho correlation coefficients were used to determine the strength of the relationships between seed predation and the analyzed site characteristics and between seed predation and seed traits. All comparisons reported used two-tailed significant tests at the 0.05 level and were performed using R [72].

## Results

### Seed removal and seed fate

With the exception of two palm seeds (an *E. edulis* seed dispersed without caching and a *S. romanzoffiana* partially buried seed, both of which were hoarded <5 cm from old-growth forest plots during the wet season), all located seeds were evidently destroyed (i.e., the endocarp was perforated, and the endosperm had been consumed). Therefore, secondary dispersal was negligible, and seed removal was referred to as seed predation, even for the eleven seeds that could not be located (<0.8%).

**Figure 1:**
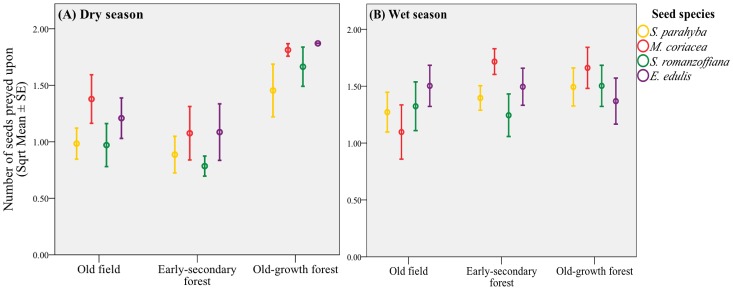
Average seed predation (square-root transformed) per seed species among habitats during the (A) dry and (B) wet seasons. Error bars indicate the standard error (SE) of the means.

The average seed predation from the 60 experimental stations was 70% (range = 61.7%–77.5%) across the two seasons. Signs of seed predation by both insects and small- and medium-bodied rodents were observed. Independent of the season, rodents played a key role as seed predators (98% of consumed seeds). A non-significant trend was determined for the relationship between morphological traits and seed predation (Spearman's correlation, P>0.05 for all comparisons): however, a post-hoc Tukey's HSD multiple comparisons test showed that the spherical-shaped and softer seeds (*M. coriacea* and *E. edulis*) were consumed more frequently than were *S. parahyba* and *S. romanzoffiana* (at P<0.05 level). Consumption by invertebrates (ants) was detected in only 2% of seeds and occurred exclusively in old field. Despite the season or seed species, old field had the lowest overall seed predation (Fig. 1).

The number of seeds attacked varied significantly with seed species (ANOVA, F = 10.3; df = 3; P<0.0001), habitat (F = 56.0; df = 2; P<0.0001) and season (F = 20.7; df = 1; P<0.0001). A significant interaction effect was determined for species×habitat×season (F = 3.7; df = 6; P = 0.001) but not for species×habitat (F = 1.85; df = 6; P = 0.08) or for species×season (F = 2.37; df = 3; p = 0.07) (Fig. 1). Surprisingly, in the dry season, the effect of habitat on seed predation was even more evident (F = 84.5; df = 2; P<0.0001). During the dry season, seed predators tended to concentrate their activity in forested habitats (Fig. 1A). The highest seed loss occurred in the wet season (Fig. 1B), being statistically greater in the old-growth and early-secondary forests than in the old field.

In the wet season, *M. coriacea* showed the same general pattern of lowest seed loss in the old field (F = 15.08; df = 2; P<0.0001), but seeds of *E. edulis, S. parahyba* and *S. romanzoffiana* were consumed equally among the habitats (P>0.05 for all comparisons, Fig. 1). Based on Tukey's HSD multiple comparisons, each individual species showed the same general pattern of highest seed predation in the old-growth forest, intermediate predation in the early-secondary forest and lowest predation in the old field during the dry season (Fig. 1).

### Seed traits, site characteristics and seed predation

**Figure 2:**
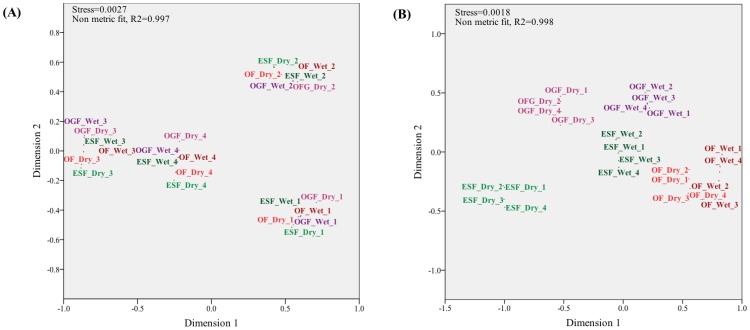
NMDS analyses of habitats (orange = Old Field; green = Early-Secondary Forest; purple = Old-Growth Forest) between seasons (dry = lighter and wet = darker colors) and among seed species (1 = *Euterpe edulis*, 2 = *Myrsine coriacea*; 3 = *Schizolobium parahyba* and 4 = *Syarus romanzoffiana*). Data were analyzed for (A) seed predation and site characteristics: LAI, DIFN, litterfall biomass, number of palm stands, and production (% FI) of ripe and unripe fleshy fruits and for (B) seed predation and morphological seed traits: seed mass, seed shape, and seed hardness (expressed as force and relative force required to crack the seed).

Non-metric multidimensional scale (NMDS) biplot projections of seed traits allow visualization of the relationship between seasonal variation and both seed predation and site characteristics, but not visualization of the relationship between seed predation and seed traits (Fig. 2). The representation of seed trait variables projected by the NMDS biplot showed four groups, one per seed species (Fig. 2A), with no seed morphological traits directly affecting seed survival (Spearman's correlation, P>0.05 for all comparisons).

The eigenvector ordination of site characteristics and seed predation resulted in five groups, which corresponded to the seasonal variance in site characteristics among habitats (Fig. 2B). Thus, notwithstanding the seed species, the ordination grouped each habitat per season. The exception was the old field grouping, which remained constant between seasons, although a slight grouping tendency was observed (Fig. 2B).

Higher seed intakes were observed in those habitats where palm trees were more abundant (Spearman's rho = 0.57; p = 0.004) and where resource availability, of both unripe and ripe fruits, was more abundant (rho = 0.44; p = 0.03 for both comparisons). Therefore, there was a positive correlation between palm stands and site fruit productivity (rho = 0.97; p<0.0001). Seed consumption was concentrated at sites with lower scores for the fraction of visible sky (DIFN; rho = −0.56; p = 0.004) and denser vegetation, which gave a higher leaf area index (LAI; rho = 0.57; p = 0.004).

## Discussion

Post-dispersal seed predators play an important role in species regeneration in Anchieta, where seeds are mostly depredated rather than dispersed; however, the rates of seed predation vary by habitat, season, and species while seed size, shape, and hardness did not affect the probability of being depredated.

In tropical early-secondary communities, the rates of seed predation assessed via seed removal experiments are usually high (50–80%) [18], [26], [50], [73], [74], while in control “pristine” areas, the rates only reach 20% [26]; however, the causes of seed mortality, as well as the influence of season and habitat on seed fate, contrast with previous findings. Differences in seed predation rates among habitats with varying vegetation cover have been previously observed in human-altered tropical forest habitats in Costa Rica [50], [75], but those differences in seed losses were attributable to insect predation (greater in mature forests), as mammal seed intake was equivalent in both early and mature forests. Ant seed predation is negligible in our study area and restricted to old field, and discrepancies in seed consumption rates among species and habitats are attributable to rodents, particularly the medium-sized agouti. The rodent community as a whole forages selectively on large-seeded species that tend to dominate annual plant biomass, however, it is expected that predation by the ant community may commonly fall most heavily on those abundant seed types [76]. The seeds used, however, were relatively large, and previous studies have shown a trend for higher ant seed removal with smaller seeds (20–100 mg; [77]).

Our findings strongly suggest that rates of seed predation by rodents vary among habitats with different logging regimes due to differences on site structure and resource distribution. In Anchieta, higher seed losses occur in areas with more dense vegetation, where fruit availability tends to be more heterogeneous and abundant [31]. This result is unexpected, given that several tropical studies have reported higher levels of seed predation in successional habitats relative to forest (e.g. [26]), [78]–[80]. The red-rumped agouti's high density (156–243 ind. km^−2^; [42]) combined with the absence of predation risk and low availability of fruits and seeds (119 kg ha^−1^y^−1^; [65]), varying both seasonally and spatially among habitats [31], may explain the intensive and indiscriminate seed predation and negligible secondary seed dispersal. Indeed, there is a general consensus that fruit scarcity alters seed handling by the scatter-hoarders, as they tend to consume (i.e., destroy) more and scatter-hoard fewer seeds when food availability is low [20], [56], [81].

Palms are considered one of the most important food species for rodents [55]–[57], [82]. Palm stands are restricted to early-secondary and old-growth forest and are more abundant in old-growth forest, a locale that exhibits a notably higher percentage of fleshy fruit in the dry season [65] and overall seed predation. In the wettest season, trees exhibit a higher percentage of individual fruiting than do palms [65], in turn causing higher rates of seed predation within all habitats. In old field, where site structural characteristics and seed predation persist steadily between seasons and seed species, seeds are predominantly attacked by small rodents [43], as indicated by teeth marks left on baited seeds. In fact, sampling of the small mammal community has shown that in Anchieta rice rats avoid microhabitats occupied by highly abundant arboreal mesopredators, including the black-eared opossum *Didelphis aurita* and coatis (*Nasua nasua*), and are more abundant in old fields [43]. Typically, these medium-sized mesopredators are abundant in the absence of top-down control [23], [83].

Our experimental results are also inconsistent with other findings showing that large-seeded species may escape predation in anthropogenic defaunated areas, while residual fauna will preferentially attack small-seeded species [28], [44], [77]. We found that the rate of seed predation by rodents was not different for small- and large-seeded species, without any morphological seed traits (seed mass, seed shape or seed hardness) being strongly linked with seed predation. Blate et al. [44] suggest that lower predation rates for large seeds may be explained by the scarcity of predators capable of penetrating their hard seed coats. Although tested on only a few species, their hypothesis could explain the greater consumption of softer-seeded species by small rodents in old field; however, this hypothesis does not apply to those habitats where agoutis are more abundant and there is often indiscriminate seed predation.

Several tropical large-seeded species are known to be strongly dependent on scatter-hoarding rodents, such as spiny rats (*Trinomys, Proechymis, Heteromys*), squirrels (*Sciurus*), acouchies (*Myoprocta*), and agoutis (*Dasyprocta*) [84], that are able to gnaw open the woody coat or disperse the seeds by scatter-hoarding in the soil, a safe site for recruitment [85], [86]; however, only seeds larger than 0.9 g are likely to be cached by agoutis [49]. In disturbed sites, where small-seeded species predominate [87], very few species have seeds large enough to escape predation by the agoutis.

In tropical forests, an increasing human-dominated fragmented and defauned environment, residual rodents are usually dominant overabundant seed predators and/or exhibit high rates of seed predation [26], [88]–[98], and might regulate the amount, location, and fate of seeds. Although rodents may have limited beneficial effects if removal sometimes results in dispersal [99], [100], for the tested Atlantic forest species, the effects appear to be predominantly negative. Howe and Brown [101] report that rodents may selectively consume and destroy seeds of some taxa more than others, thereby providing a competitive advantage to less preferred species. This suggests in predator-free habitats, the resilient rodent community may represent a severe bottleneck in regeneration process, with a potential and significant role in biodiversity loss and lowered carrying capacity for consumers [23]. Habitat fragmentation and/or isolation could also alter the relevance of interactions that could affect biodiversity in many ways [102]. These results have profound implications for the conservation and restoration of increasingly disturbed and impoverished tropical habitats.
